# Utilization of potentially inappropriate sedative-hypnotic and atypical antipsychotic medications among elderly individuals with insomnia and Alzheimer’s disease

**DOI:** 10.1093/sleep/zsaf003

**Published:** 2025-01-25

**Authors:** Farid Chekani, Kirti Mirchandani, Saba Zaki, Swarnali Goswami, Manvi Sharma

**Affiliations:** Merck & Co., Inc., Rahway, NJ, USA; Complete HEOR Solutions (CHEORS), Chalfont, PA, USA; Complete HEOR Solutions (CHEORS), Chalfont, PA, USA; Complete HEOR Solutions (CHEORS), Chalfont, PA, USA; Complete HEOR Solutions (CHEORS), Chalfont, PA, USA

**Keywords:** insomnia, aged, potentially inappropriate medication list, hypnotics and sedatives

## Abstract

**Study Objectives:**

This study assessed the utilization of potentially inappropriate medications (PIM) including oral sedative-hypnotic and atypical antipsychotic (OSHAA), healthcare resource utilization (HCRU), and costs among elderly individuals with insomnia and in the subpopulation with Alzheimer’s disease (AD) who also had a diagnosis of insomnia.

**Methods:**

Using a claims database containing International Classification of Diseases, 10th Revision (ICD-10) codes, the cohort included individuals aged ≥ 65 with incident insomnia (EI, *N* = 152 969) and AD insomnia subpopulation (ADI, *N* = 4888). The proportion of patients utilizing atypical antipsychotics or oral sedative-hypnotic medications, namely z-drugs, benzodiazepines, doxepin, dual orexin receptor antagonists (DORAs), and melatonin agonists, were assessed. Inappropriate OSHAA utilization was defined as per the American Geriatrics Society (AGS) Beers criteria. Multivariable models were utilized to compare HCRU and costs between PIM-OSHAA and no PIM-OSHAA groups.

**Results:**

Among the EI cohort, z-drugs (13.39%) were the most commonly utilized OSHAA, and in the ADI cohort, it was AAPs (29.97%). PIM-OSHAA was utilized by 20% of the EI and 35% of the ADI cohorts. Patients with PIM-OSHAA use among the EI cohort had a higher annualized adjusted mean HCRU (pharmacy visits: 31.21 vs. 23.68; ambulatory and outpatient visits: 18.55 vs. 16.85) and costs, primarily due to medical costs (mean total cost: $36 676.08 vs. $31 346.54) compared to those without.

**Conclusions:**

Substantial utilization of PIM-OSHAA was observed in EI and ADI cohorts. PIM-OSHAA use was associated with higher HCRU and costs. These findings underscore the importance of appropriate treatment strategies for insomnia in the elderly population especially in those with concurrent AD.

## Graphical Abstract

**Graphical Abstract:**
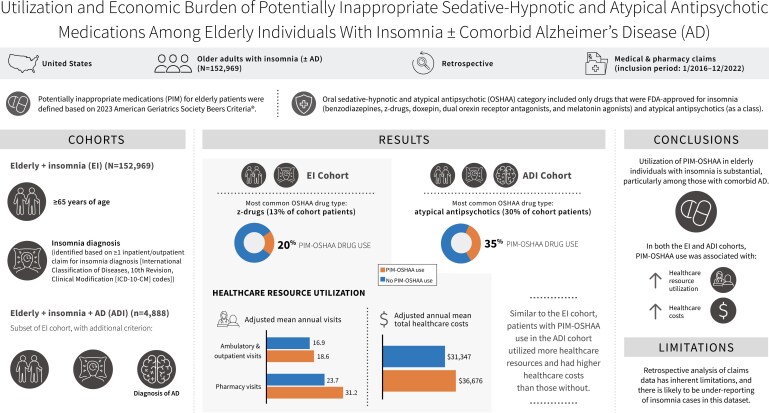

**Statement of Significance**

In older adults, pharmacological treatment for insomnia is an important area of research due to the aging population and the risks involved with the utilization of sedative-hypnotic medications. In this study, potentially inappropriate use of oral sedative-hypnotic medications was defined based on well-established criteria provided by the American Geriatric Society and was assessed for older adults with insomnia and Alzheimer’s disease. The associated healthcare resource utilization and costs were also evaluated.

## Introduction

Insomnia, as stated by the Diagnostic and Statistical Manual of Mental Disorders (DSM-V), is characterized by dissatisfaction with sleep quantity or quality, persisting for at least 3 months, and associated with difficulty initiating or maintaining sleep, early-morning awakenings, or sleep disturbances [1]. Insomnia is a common disorder of aging, with symptoms prevalent in 30% to 48% of the elderly [2]. Moreover, aging is a risk factor for both insomnia and Alzheimer’s disease (AD). Thus, sleep disorders are notably prevalent in patients with AD [1].

Pharmacological management of insomnia often involves oral sedative-hypnotic medications, encompassing benzodiazepines, benzodiazepine derivatives (also referred to as z-drugs), dual orexin receptor antagonists (DORAs), and other agents, such as doxepin and melatonin [3]. Atypical antipsychotics (AAPs) exhibit sedative effects and are commonly prescribed off-label for sedation in patients with chronic insomnia [3–5]. The American Geriatrics Society (AGS) Beers criteria (2023) offer detailed insights into potentially inappropriate medications (PIMs) for elderly individuals and offer guidelines that are crucial for improving care and outcomes in vulnerable populations [6]. While oral sedative-hypnotic and atypical antipsychotic (OSHAA) medications have demonstrated efficacy in reducing sleep latency and improving sleep efficiency, PIMs can lead to adverse outcomes in elderly patients. AAPs and most sedative-hypnotic medications are considered PIMs for elderly individuals, as per AGS Beers criteria (2023) [2, 6]. Some adverse effects of PIM-OSHAA medications include impaired coordination, somnolence, and balance issues [6]. It is important to note that DORAs are not considered as PIMs, as per the AGS Beers Criteria 2023, hence deemed appropriate [6]. The prescription of appropriate medications for insomnia management is crucial for patients.

To our knowledge, limited database studies have evaluated hypnotic use among elderly patients with insomnia considering AGS beers criteria. A claims-based study compared benzodiazepines versus low-dose trazodone in terms of HCRU and costs and found negative impacts associated with benzodiazepine use [7]. Some observational studies have assessed the use of inappropriate medications in the elderly population but did not focus on insomnia treatment [8, 9]. More research is needed to understand OSHAA utilization patterns and associated economic outcomes among elderly patients with insomnia, especially among those with AD [2, 3]. To address these research gaps, this study aimed to describe OSHAA medication utilization and compare the all-cause healthcare resource utilization (HCRU) and costs between patients with and without potentially inappropriate OSHAA medications (PIM-OSHAA) use, among elderly patients with insomnia (EI) and in the subpopulation of patients with AD (ADI). The results of this study provide insights into the burden of PIM-OSHAA utilization in elderly patients with insomnia. This study utilized the AGS Beers criteria to identify PIM-OSHAA, enhancing comprehension of how medication choices impact economic outcomes in the elderly population [6].

## Methods

### Study design and data source

**Figure 1.**
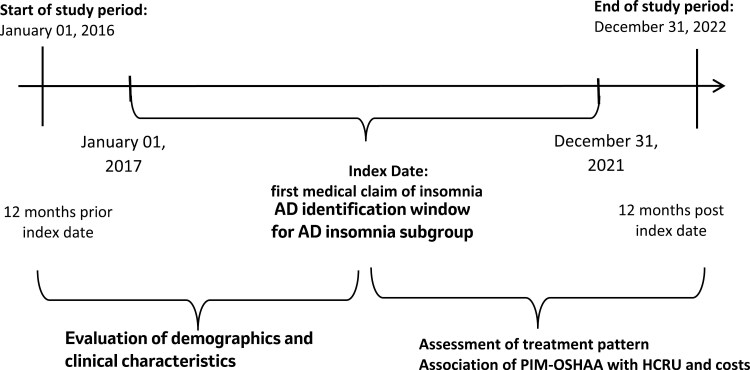
Study Schematic. Notes: AD: Alzheimer’s disease, HCRU: Healthcare resource utilization; PIM-OSHAA: Potentially inappropriate oral sedative-hypnotic and atypical antipsychotic medication.

This retrospective cohort study utilized de-identified US healthcare administrative claims data from MERATIVE MarketScan Commercial Claims and Encounters (CCAE) and Medicare Supplemental Database [10]. This database consists of medical and pharmacy claims data for over 215 million individuals, enrolled in employer-sponsored private health insurance and Medicare-covered retirees with employer-sponsored supplemental insurance. This study used medical and pharmacy claims between January 1, 2016 and December 31, 2022. International Classification of Diseases, 10th Revision, Clinical Modification (ICD-10-CM) codes, and National Drug Codes (NDC) were utilized to identify relevant diagnoses including insomnia (Supplementary Table 1) and treatments (Supplementary Table 2). In this study, medical diagnoses were derived from administrative claims data. These diagnostic codes were neither derived from clinical research interviews nor validated against chart review. Figure 1 illustrates the study design.

### Study participants

The analysis included individuals aged 65 years and above with at least one inpatient or outpatient medical claim for insomnia diagnosis, during the index period (January 1, 2017 to December 31, 2021). The earliest date of medical claim with an insomnia diagnosis was defined as the index date. To be considered for the study, patients without a history of insomnia diagnosis during 12 months prior to the index date were included. The rationale for selecting incident cases was to avoid prevalence-incidence bias. Individuals with a gap in pharmacy, medical, or mental health coverage enrollment for more than 45 days, during 12 months prior (baseline) and 12 months post (follow-up) index date, were excluded [11]. In addition, patients with a history of schizophrenia, bipolar disorder (Supplementary Table 1), and non-insomnia sleep disorder (Supplementary Table 3) during the baseline period, were also excluded [12]. The rationale for excluding schizophrenia, bipolar disorder, and other psychotic disorders was related to missing claims data associated with mental health service utilization which is common in these chronic psychotic conditions. Patients with insomnia fulfilling the inclusion and exclusion criteria specified above were categorized as the elderly with insomnia (EI) cohort. Among the EI cohort, the subpopulation with an AD diagnosis in the 12 months preceding or following the index date was identified as the elderly with AD and insomnia (ADI) cohort. AD diagnosis was identified based on ICD-10 codes which might not be specific enough to differentiate AD from other types of dementia. Thus, the ADI cohort might include patients with possible AD or Dementia with Lewy Bodies under a broader category of Alzheimer’s disease and related dementias (ADRD).

### Measures

Oral sedative-hypnotic medications were identified based on the list of “prescription insomnia drugs” provided by the Food and Drug Administration, namely z-drugs (zopiclone, eszopiclone, zaleplon, and zolpidem), benzodiazepines (estazolam, flurazepam, quazepam, temazepam, and triazolam), doxepin, DORAs (daridorexant, lemborexant, and suvorexant), and melatonin agonist (ramelteon and tasimelteon) [13]. In addition to the FDA list, AAPs were also assessed in this study as a drug class, given their sedative-hypnotic properties and the fact that AAPs are considered high-risk medications for elderly individuals [5, 14, 15]. No other off-label sedative-hypnotic medications were included in the analysis. These treatments were identified using NDC codes (Supplementary Table 2). AGS Beers criteria (2023) was utilized to categorize patients with OSHAA utilization into PIM-OSHAA and no PIM-OSHAA groups. AAPs were classified under PIM-OSHAA as per the AGS Beers criteria [6]. Other OSHAAs included in the PIM-OSHAA group were z-drugs, benzodiazepines, and doxepin (> 6 mg/day). No PIM-OSHAA group included FDA-approved OSHAA medications that were not listed in the AGS Beers criteria, such as DORAs, doxepin (≤ 6 mg/day), and melatonin agonists, and patients using OSHAA medications which are deemed appropriate or those not using any of the medications of interest.

Covariates included in this analysis were age group, sex, region, insurance plan type, index year, and clinical characteristics, including Elixhauser Comorbidity Index (ECI) and behavioral or neuropsychiatric symptoms (NPS) identified during the baseline period [16]. The identification of behavioral symptoms was based on definitions used in published literature and included agitation/aggression (agitation/aggression, irritability, wandering, and sexual disinhibition), psychotic symptoms (delusions and hallucinations), and anxiety/mood disorders (including anxiety, mood disorders, apathy, and depression) [17–26]. Behavioral symptoms and other comorbidities were identified using ICD-10-CM diagnosis codes (Supplementary Table 1).

The following categories of all-cause HCRU were assessed in the follow-up period: inpatient length of stay (LOS) (in days), inpatient visits, ambulatory care and outpatient visits, emergency room (ER) visits, physician office visits, pharmacy visits, and other medical claims. All-cause costs for these HCRU components were calculated from the paid amounts of fully adjudicated claims, including net payments (NETPAY) made by the primary payer and the Coordination of benefits, which is the amount secondary insurance pays. Total costs were calculated as the sum of medical and pharmacy costs borne by the payer. All costs were reported as per person per year (PPPY) and were adjusted to 2023 US dollars using the medical care component of the Consumer Price Index.

### Statistical analysis

Descriptive statistics were used to summarize patient characteristics among EI and ADI cohorts, categorized by PIM-OSHAA use. The categorical variables were presented as frequencies (percentages), and statistical significance was assessed using Chi-square tests. The continuous variables were presented as mean (standard deviation [SD]) and statistical significance was assessed using Student’s *t*-tests.

The proportion of elderly patients utilizing OSHAA medications was obtained by dividing the number of patients with at least one pharmacy claim for each OSHAA medication of interest during the follow-up period by the total number of patients included in the cohort. The proportion of patients with and without PIM-OSHAA use was also described.

Separate multivariable generalized linear models (GLM) were fitted to compare each HCRU and cost outcome, adjusting for the covariates listed in the measures section. The primary explanatory variable of interest was the PIM-OSHAA use. For those where more than 30% of the patients had a value of zero for the outcome, a two-part model was employed [27]. The first part of the two-part model estimated the odds ratio (OR) (95% confidence interval [CI]) (using a binary logit model) of having a positive HCRU or cost. The second part estimated the incidence rate ratio (IRR) for those with positive HCRU or costs. For all the other HCRU and cost outcomes, a one-part model was used, and the IRR (95% CI) was reported. For the GLM, the appropriate distribution and link were selected based on the Modified Park test [28]. The models employed gamma distribution to calculate costs. The models for HCRU used either Poisson, negative binomial, or gamma distributions. All analyses in this study were done using SAS 9.4 (Cary, NC). The α level threshold was 0.05 for all the statistical tests.

In addition, a sensitivity analysis was done using inverse probability of treatment weighting (IPTW) to compare HCRU and costs during the follow-up period among patients with and without PIM-OSHAA use in the EI and ADI cohorts [29]. The IPTW method was employed to mitigate the impact of observed confounders. The weights were calculated using propensity scores for being prescribed PIM-OSHAA among EI and ADI cohorts separately. Propensity scores were estimated using a logistic regression model with PIM-OSHAA use as the dependent variable and potential confounders listed in the measures section as the independent variables. Following IPTW, covariate balance was assessed in the weighted sample using the standardized mean difference with a threshold of 0.1.

## Results

### Selection of the study sample

**Figure 2.**
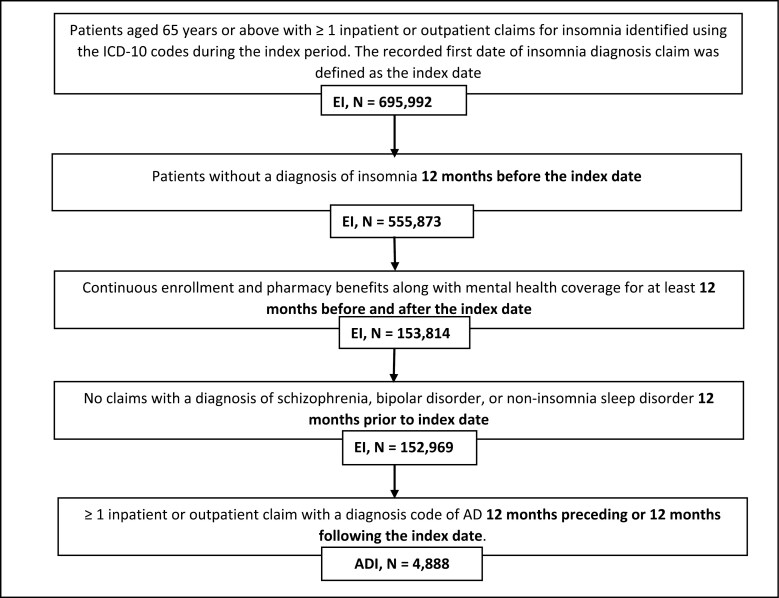
Cohort selection diagram. Notes: AD: Alzheimer’s disease; ADI: Elderly with Alzheimer’s disease and insomnia; EI: Elderly with insomnia; ICD: International Classification of Diseases.

This study identified 695 992 patients aged 65 years and above with ≥ 1 inpatient or outpatient claim for insomnia. After applying all the inclusion and exclusion criteria, 152 969 patients in the EI cohort and 4888 patients in the ADI cohort were identified (Figure 2).

### Baseline demographics and clinical characteristics

The characteristics of patients with and without PIM-OSHAA use among the EI and ADI cohorts were similar. In the EI cohort, a mean age of 73.7 versus 73.4 years, and primarily females (60.21% vs. 51.74%) were observed (Table 1). Similarly, among the ADI cohort, a mean age of 83.1 years versus 83.9 years and predominantly females (59.14% vs. 59.53%) were observed (Table 2). Notably, among both cohorts, patients with PIM-OSHAA use had a higher proportion of NPS, most commonly anxiety and mood disorders compared to those without (EI: 33.26% vs. 23.44%; ADI: 53.47% vs. 43.73%). Additional characteristics for EI and ADI cohorts are described in Supplementary Tables 4 and 5, respectively.

**Table 1.** Baseline characteristics of patients with or without PIM-OSHAA use among EI cohort

Description	PIM-OSHAA	No PIM-OSHAA	*P-*value
	(*N* = 30 705)	(*N* = 122 264)	
**Age at index date**			
Mean (SD)	73.7 (8.12)	73.4 (7.91)	<.001*
**Age group at index date *N* (%)**			
65–69	12 109 (39.44%)	49 799 (40.73%)	<.001*
70–74	6503 (21.18%)	25 863 (21.15%)	
75–79	4902 (15.96%)	19 850 (16.24%)	
80–84	3380 (11.01%)	13 043 (10.67%)	
85+	3811 (12.41%)	13 709 (11.21%)	
**Sex *N* (%)**			
Female	18 489 (60.21%)	63 259 (51.74%)	<.001*
Male	12 216 (39.79%)	59 005 (48.26%)	
**ECI**			
Mean (SD)	4.2 (4.71)	4.4 (4.70)	<.001*
**NPS sub-types** ***N* (%)**			
NPS overall	10 501 (34.20%)	29 130 (23.83%)	<.001*
Psychotic symptoms	811 (2.64%)	817 (0.67%)	<.001*
Agitation/Aggression	350 (1.14%)	429 (0.35%)	<.001*
Anxiety and mood disorders	10 212 (33.26%)	28 653 (23.44%)	<.001*

EI: Elderly with insomnia; ECI: Elixhauser Comorbidity Index; PIM-OSHAA: Potentially inappropriate oral sedative hypnotics and atypical antipsychotics; NPS: Neuropsychiatric symptoms SD: Standard deviation.

^*^ *p*-Value <.05. Significant *p-*values are marked as bold.

**Table 2.** Baseline characteristics of patients with or without PIM-OSHAA use among ADI cohort

Description	PIM-OSHAA	No PIM-OSHAA	*P*-value
	(*N* = 1728)	(*N* = 3160)	
**Age at index date**			
Mean (SD)	83.1 (7.66)	83.9 (7.76)	.001*
**Age group at index date *N* (%)**			
65–69	94 (5.44%)	147 (4.65%)	.091
70–74	148 (8.56%)	237 (7.50%)	
75–79	296 (17.13%)	500 (15.82%)	
80–84	390 (22.57%)	689 (21.80%)	
85+	800 (46.30%)	1587 (50.22%)	
**Sex *N* (%)**			
Female	1022 (59.14%)	1881 (59.53%)	.795
Male	706 (40.86%)	1279 (40.47%)	
**ECI**			
Mean (SD)	6.2 (4.48)	6.6 (4.52)	<.001*
**NPS sub-types** ***N* (%)**			
NPS overall	1048 (60.65%)	1482 (46.90%)	<.001*
Psychotic symptoms	297 (17.19%)	188 (5.95%)	<.001*
Agitation/Aggression	191 (11.05%)	119 (3.77%)	<.001*
Anxiety and mood disorders	924 (53.47%)	1382 (43.73%)	<.001*

ADI: Elderly with Alzheimer’s disease and insomnia; ECI: Elixhauser Comorbidity Index; PIM-OSHAA: Potentially inappropriate oral sedative hypnotics and atypical antipsychotics; NPS: Neuropsychiatric symptoms SD: Standard deviation.

^*^ *p-*Value <.05.. Significant *p-*Values are marked as bold.

### Utilization of OSHAA and PIM-OSHAA

**Figure 3.**
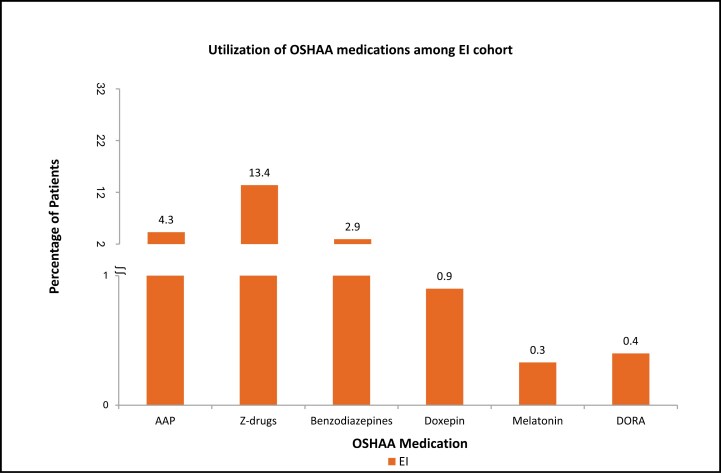
Proportion of patients with OSHAA medications among EI in the follow-up period. Notes: AAP: Atypical antipsychotic; Benzodiazepines; DORA: Dual orexin receptor antagonist; EI: Elderly with insomnia; OSHAA: Oral sedative-hypnotic and atypical antipsychotic; Z-drugs: Non-benzodiazepines.

**Figure 4.**
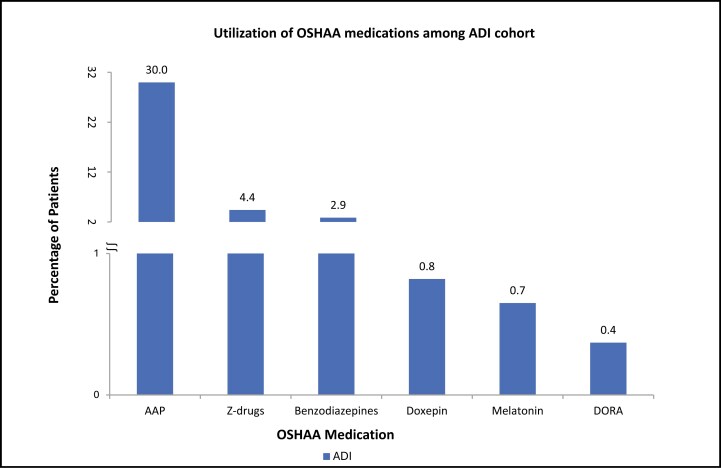
Proportion of patients with OSHAA medications among the ADI cohort in the follow-up period. Notes: AAP: Atypical antipsychotic; ADI: Elderly with Alzheimer’s disease and insomnia; Benzodiazepines; DORA: Dual orexin receptor antagonist; OSHAA: Oral sedative-hypnotic and atypical antipsychotic; Z-drugs: Non-benzodiazepines.

Among the EI cohort, the proportion of elderly patients with prescribed OSHAA medications was as follows: z-drugs (13.39%) followed by AAPs (4.29%) and benzodiazepines (2.94%). A lower proportion of patients received prescriptions for melatonin (0.33%) and DORAs (0.40%) (Figure 3). Among the ADI cohort, the proportion of the patients with prescribed OSHAA medications was as follows: AAPs (29.97%) followed by z-drugs (4.40%) and benzodiazepines (2.86%). A lower proportion of patients were prescribed DORAs (0.37%), followed by melatonin (0.65%) (Figure 4). Around 20% of the EI cohort and around 35% of the ADI cohort were prescribed PIM-OSHAA medications.

### Comparison of HCRU and healthcare costs

#### EI cohort.

Patients with PIM-OSHAA use had higher HCRU as compared to patients without, especially in the following categories: 18% longer inpatient stay (IRR [95% CI]: 1.18 [1.15,1.21]) and 27% more pharmacy visits (IRR [95% CI]: 1.27 [1.27,1.28]) (Table 3). Patients with PIM-OSHAA use had 13% more total costs (medical + pharmacy) (IRR [95% CI]: 1.13 [1.11,1.15]) than patients without. In addition, these patients had 18% more pharmacy costs (IRR [95% CI]: 1.18 [1.16,1.21]) and 10% more physician costs (IRR [95% CI]: 1.10 [1.07,1.13]) (Table 4).

**Table 3.** Comparison of all-cause HCRU between patients with and without PIM-OSHAA use: EI cohort

Categories	OR [95% CI]	*P-*value	IRR [95% CI]	*P-*value
Inpatient LOS (in days)	1.15 [1.12,1.19]	**<.001**	1.18 [1.15,1.21]	<.001*
Inpatient visits	1.15 [1.11,1.19]	**<.001**	1.04 [1.03,1.05]	<.001*
Ambulatory care and outpatient visits	NA		1.07 [1.06,1.08]	<.001*
Other medical claims	NA		1.00 [0.99,1.02]	.924
ER visits	1.11 [1.08,1.15]	**<.001**	1.10 [1.09,1.12]	<.001*
Physician visits	NA		1.02 [1.01,1.04]	<.001*
Pharmacy visits	NA		1.27 [1.27,1.28]	<.001*

CI: Confidence interval; EI: Elderly with insomnia; ER: Emergency room; IRR: Incidence rate ratio; HCRU: Healthcare resource utilization; LOS: Length of stay; OR: Odds ratio; PIM-OSHAA: Potentially inappropriate oral sedative hypnotics and atypical antipsychotics; NA: Not applicable, as one part model was used (see Statistical Analysis).

^*^ *p*-Value <0.05. OR and IRR are for patients with PIM-OSHAA use relative to those without. *p-*values less than 0.05 are significant and are marked in bold.

**Table 4.** Comparison of all-cause healthcare costs between patients with and without PIM-OSHAA use: EI cohort

Categories	OR [95% CI]	*P-*value	IRR [95% CI]	*P-*value
Total costs	NA		1.13 [1.11,1.15]	<.001*
Inpatient costs	1.15 [1.11,1.19]	**<.001**	1.05 [1.01,1.08]	.005*
Ambulatory Care & Outpatient costs	NA		1.09 [1.07,1.11]	<.001*
Other medical claims costs	NA		1.12 [1.08,1.15]	<.001*
ER costs	1.12 [1.08,1.15]	**<.001**	1.11 [1.08, 1.14]	<.001*
Physician costs	NA		1.10 [1.07,1.13]	< .001*
Pharmacy costs	NA		1.18 [1.16,1.21]	<.001*

CI: Confidence interval; EI: Elderly with insomnia; ER: Emergency room; IRR: Incidence rate ratio; HCRU: Healthcare resource utilization; OR: Odds ratio; PIM-OSHAA: Potentially inappropriate oral sedative hypnotics and atypical antipsychotics; NA: Not applicable, as one part model was used (see Statistical Analysis).

^*^ *p*-Value <.05. OR and IRR are for patients with PIM-OSHAA use relative to those without. *p-*Values less than .05 are significant and are marked in bold.

**Figure 5.**
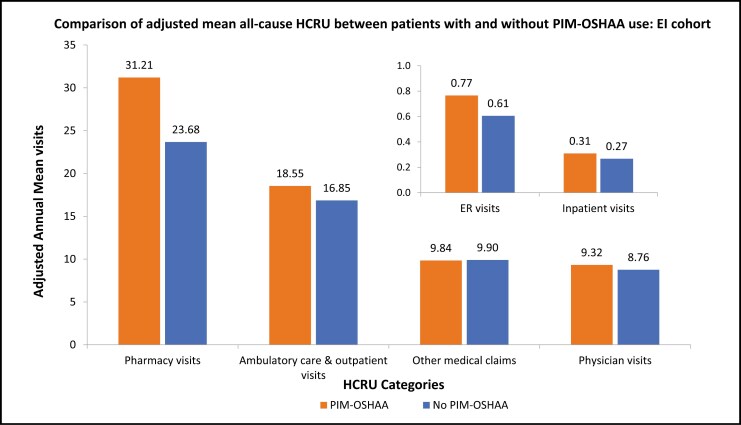
Comparison of adjusted mean all-cause HCRU between patients with or without PIM-OSHAA use among EI cohort. Notes: EI: Elderly with insomnia; ER: Emergency room; HCRU: Healthcare resource utilization; PIM-OSHAA: Potentially inappropriate oral sedative-hypnotic and atypical antipsychotic medication.

**Figure 6.**
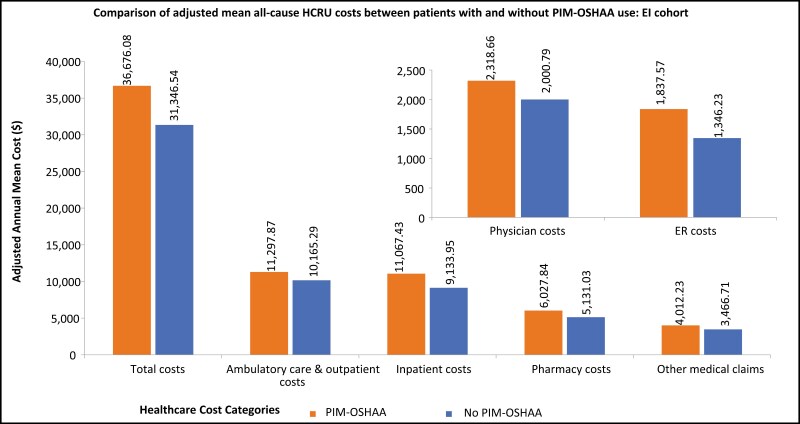
Comparison of adjusted mean all-cause costs between patients with or without PIM-OSHAA use among EI cohort. Notes: EI: Elderly with insomnia; ER: Emergency room; HCRU: Healthcare resource utilization; PIM-OSHAA: Potentially inappropriate oral sedative-hypnotic and atypical antipsychotic medication.

Patients with PIM-OSHAA use had higher adjusted annual mean visits corresponding to the pharmacy (mean [SD]: 31.21 [7.91] vs. 23.68 [5.72]), ambulatory care and outpatient (mean [SD]: 18.55 [6.79] vs. 16.85 [6.00]), physician (mean [SD]: 9.32 [2.15] vs. 8.76 [2.04]), ER (mean [SD]: 0.77 [0.48] vs. 0.61 [0.37]), and inpatient (mean [SD]: 0.31 [0.21] vs. 0.27 [0.18]) compared to patients without (Figure 5). Patients with PIM-OSHAA use had higher adjusted annual mean total healthcare costs (mean [SD]: $36,676.08 [$28,395.35] vs. $31,346.54 [$23,459.84]) compared to patients without. The following cost categories made significant contributions to the total costs: ambulatory care and outpatient costs (mean [SD]: $11,297.87 [$8,769.28] and $10,165.29 [$7,706.86], among patients with and without PIM-OSHAA group, respectively), followed by inpatient costs (mean [SD]: $11,067.43 [$8,297.56] and $9,133.95 [$7,048.88], among patients with and without PIM-OSHAA group, respectively), and pharmacy costs (mean [SD]: $6,027.84 [$4,039.42] and $5,131.03 [$3,247.51], among patients with and without PIM-OSHAA group, respectively) (Figure 6).

#### ADI cohort.

Similar to the EI cohort, patients with PIM-OSHAA use in the ADI cohort utilized more healthcare resources and had higher healthcare costs than those without. Patients with PIM-OSHAA use had 20% longer inpatient stays (IRR [95% CI]: 1.20 [1.10,1.30]) and 17% more other medical claims (IRR: 1.17 [1.10,1.25]) (Table 5). Patients with PIM-OSHAA use had 14% more total costs (medical + pharmacy) (IRR [95% CI]: 1.14 [1.06,1.22]) than compared to those without (Table 6).

**Table 5.** Comparison of all-cause HCRU between patients with or without PIM-OSHAA use among ADI cohort

Categories	OR [95% CI]	*P-*value	IRR [95% CI]	*P-*value
Inpatient LOS (in days)	1.55 [1.37,1.76]	**<.001**	1.20 [1.10,1.30]	**<.001**
Inpatient visits	1.52 [1.34,1.73]	**<.001**	1.06 [1.02,1.10]	**.007**
Ambulatory Care & Outpatient visits	NA		1.06 [1.02,1.11]	**.002**
Hospice visits	0.95 [0.51,1.78]	.870	0.77 [0.51,1.17]	.221
Other medical claims	NA		1.17 [1.10,1.25]	**<.001**
ER visits	1.57 [1.38,1.77]	**<.001**	1.14 [1.07,1.21]	**<.001**
Physician visits	NA		0.97 [0.91,1.04]	.451
Pharmacy visits	NA		1.16 [1.12,1.20]	**<.001**

ADI: Elderly with Alzheimer’s disease and insomnia; CI: Confidence interval; ER: Emergency room; IRR: Incidence rate ratio; HCRU: Healthcare resource utilization; LOS: Length of stay; OR: Odds ratio; PIM-OSHAA: Potentially inappropriate oral sedative hypnotics and atypical antipsychotics; NA: Not applicable, as one part model was used (see Statistical Analysis).

^*^OR and IRR are for patients with PIM-OSHAA use relative to those without. *p-*Values less than .05 are significant and are marked in bold.

**Table 6.** Comparison of all-cause healthcare costs between patients with or without PIM-OSHAA use among ADI cohort

Categories	OR [95% CI]	*P-*value	IRR [95% CI]	*P-*value
Total costs	NA		1.14 [1.06,1.22]	<.001*
Inpatient costs	1.53 [1.35,1.74]	**<.001**	1.04 [0.94,1.14]	.498
Ambulatory Care & Outpatient costs	NA		0.92 [0.86,0.99]	.019*
Hospice costs	1.55 [1.37,1.75]	**<.001**	0.64 [0.63, 0.64]	<.001*
Other medical claims costs	NA		1.03 [0.90,1.17]	.696
ER costs	1.55 [1.37,1.76]	**<.001**	1.08 [0.97, 1.20]	.138
Physician costs	NA		0.87 [0.76,1.00]	.049*
Pharmacy costs	NA		1.06 [0.96,1.16]	.234

ADI: Elderly with Alzheimer’s disease and insomnia; CI: Confidence interval; ER: Emergency room; IRR: Incidence rate ratio; HCRU: Healthcare resource utilization; OR: Odds ratio; PIM-OSHAA: Potentially inappropriate oral sedative hypnotics and atypical antipsychotics; NA: Not applicable, as one part model was used (see Statistical Analysis).

^*^ *p*-Value <.05. OR and IRR are for patients with PIM-OSHAA use relative to those without. *p-*values less than .05 are significant and are marked in bold.

**Figure 7.**
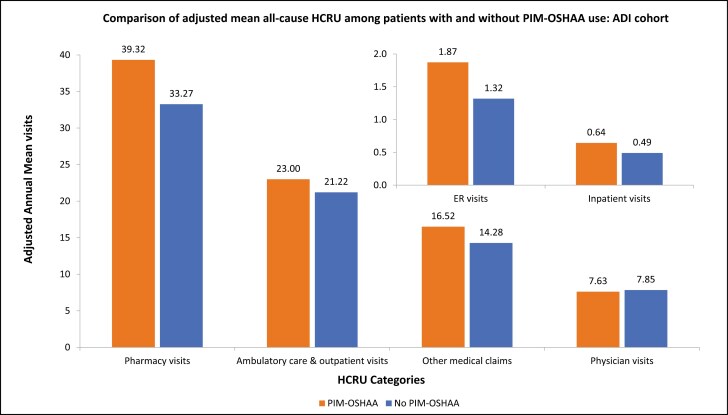
Comparison of adjusted mean all-cause HCRU between patients with or without PIM-OSHAA use: ADI cohort. Notes: ADI: Elderly with Alzheimer’s disease and insomnia; ER: Emergency room; HCRU: Healthcare resource utilization; PIM-OSHAA: Potentially inappropriate oral sedative-hypnotic and atypical antipsychotic medication.

**Figure 8.**
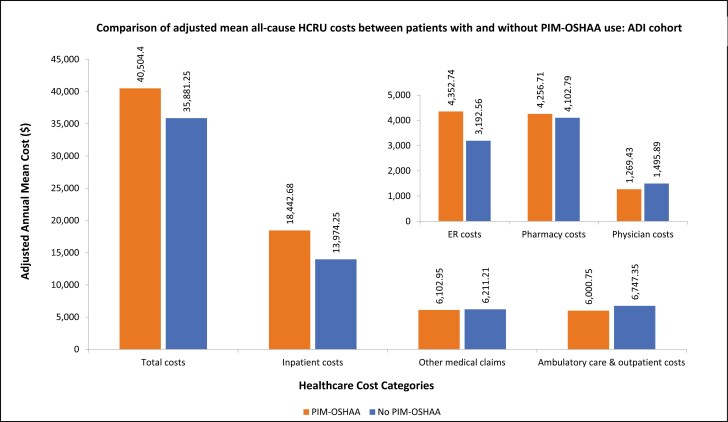
Comparison of adjusted mean all-cause healthcare costs between patients with or without PIM-OSHAA use among ADI cohort. Notes: ADI: Elderly with Alzheimer’s disease and insomnia; ER: Emergency room; HCRU: Healthcare resource utilization; PIM-OSHAA: Potentially inappropriate oral sedative-hypnotic and atypical antipsychotic medication.

Patients with PIM-OSHAA use had higher adjusted annual mean visits corresponding to the pharmacy (mean [SD]: 39.32 [7.29] vs. 33.27 [5.95]), ambulatory care and outpatient (mean [SD]: 23 [5.38] vs. 21.22 [4.88]), other medical claims (mean [SD]: 16.52 [5.02] vs. 14.28 [4.49]), ER (mean [SD]: 1.87 [0.56] vs. 1.32 [0.42]), inpatient LOS (mean [SD]: 4.63 [1.72] vs. 2.99 [1.23]), and inpatient (mean [SD]: 0.64 [0.18] vs. 0.49 [0.16]) compared to those without (Figure 7). Patients with PIM-OSHAA use had higher adjusted annual mean total healthcare costs (mean [SD]: $40,504.41 [$20,542.72] vs. $35,881.25 [$18,611.46]), compared to patients without PIM-OSHAA use. The following cost categories made significant contributions to the total costs: inpatient costs (mean [SD]: $18,442.68 [$11,503.16] and $13,974.25 [$9,317.43], among patients with and without PIM-OSHAA group, respectively) followed by other medical claims costs (mean [SD]: $6,102.95 [$4,140.17] and $6,211.21 [$4,363.72], among patients with and without PIM-OSHAA group, respectively), and ambulatory care and outpatient costs (mean [SD]: $6,000.75 [$3,986.19] and $6,747.35 [$4,631.84], among patients with and without PIM-OSHAA group, respectively) (Figure 8).

For the sensitivity analysis, after applying the IPTW to the study cohorts, the covariates in the two groups were balanced [30]. Adjusted models employing IPTW-generated estimates were similar to the primary analysis findings of comparing HCRU and healthcare costs between patients with and without PIM-OSHAA use among the EI (Supplementary Table 6, Supplementary Figure 1, and Supplementary Figure 2) and ADI cohorts (Supplementary Table 7, Supplementary Figure 3, and Supplementary Figure 4).

## Discussion

In this retrospective study, PIM-OSHAA use was common among EI in the United States and was associated with increased HCRU and costs. PIM-OSHAAs were prescribed to one-fifth of the EI cohort and approximately one-third of the ADI cohort. Among the EI and ADI cohorts, patients with PIM-OSHAA use had higher HCRU across the various categories than those without PIM-OSHAA use. Total healthcare costs were higher for patients prescribed PIM-OSHAA than those without, which was observed to be related primarily to medical costs followed by pharmacy costs. Given the limited literature evaluating the utilization and association of PIM-OSHAAs with healthcare burden among the elderly with insomnia and those with comorbid AD, our study fills an important evidence gap.

The characteristics of our study cohort are consistent with those of elderly patients with insomnia identified in published literature [8, 9]. The patients included in our study had a mean age of ~73 years and were predominantly female, with a high ECI score. It was observed that the patients with PIM-OSHAA use had a significantly higher number of NPS, most commonly anxiety and mood disorders, as compared to those without PIM-OSHAA use among both cohorts. These results are consistent with the findings presented by Dragioti et al., which highlighted that insomnia severity is associated with a high risk of anxiety and mood disorders, although their study focused on elderly individuals with pain [31]. A study by Benoit et al. presented a similar finding that anxiety was the most common NPS among elderly with AD [32]. Moreover, a registry-based study corroborating our findings suggested that increased PIM-OSHAA use is common among the elderly with multiple NPS and predisposed major neurocognitive disorder [33]. Further studies are needed to understand the impact of PIM-OSHAA use on the longitudinal patterns of NPS among elderly patients with insomnia.

Our study assessed the treatment patterns of OSHAA medications among EI and ADI cohorts. A broader definition of sedative-hypnotics can include all medications that cause central nervous system (CNS) depression. However, this study focused on those sedative-hypnotics that are approved by FDA for insomnia treatment in the United States, in addition to AAPs as a drug class. With this rationale, off-label use of many CNS depressants was not taken into account as insomnia treatment. We would recommend further research using a broader definition of sedative-hypnotics to include commonly prescribed medications for insomnia such as trazodone, mirtazapine, amitriptyline, and nortriptyline as well as some benzodiazepines such as lorazepam, clonazepam, and diazepam.

The study results indicated that among the EI cohort, a substantial proportion of the patients were prescribed z-drugs (13.39%), followed by AAPs (4.29%), with a low proportion of patients receiving DORAs (0.40%). Wickwire et al. assessed elderly patients with insomnia who were most commonly prescribed zolpidem (78.6%), followed by doxepin (6.2%), and eszopiclone (5.5%) [34]. Differences in study findings can be attributed to the data source, study design and methodology, and definition of insomnia. The comparative study identified the patients with insomnia using only prescribed sedative-hypnotics, whereas our study focused on patients with diagnosed insomnia, identified using ICD-10-CM codes in claims data, which is consistent with published literature [19, 35]. Limited information exists regarding the use of OSHAA medications among patients with insomnia and comorbid AD. Our findings are supported by the findings from the Machado-Duque et al. study, which reported that most patients with AD were prescribed AAP, which is potentially inappropriate because it may exacerbate cognitive impairment [36].

Among the EI cohort, it was found that 20.07% of patients had evidence of PIM-OSHAA use. Pek et al., assessing the institutionalized elderly population taking newly prescribed benzodiazepine or sedative-hypnotics, highlighted that 15.9% of patients had at least 1 PIM [37]. The differences in study findings could be attributed to the study population and the sedative-hypnotic drugs analyzed, which included all benzodiazepines, non-benzodiazepines, and zopiclone, whereas, in our study, additional insomnia medications (AAPs, DORAs, and melatonin agonists) were included [9]. Another study assessing community-dwelling Medicare beneficiaries found that 29% of the beneficiaries were taking one or more PIMs, predominantly associated with insomnia. The relatively lower proportion of patients with PIM-OSHAA use in our study could be because our study focused on the assessment of only inappropriate sedative-hypnotic medications and not all the PIMs.

For the ADI cohort, 35.35% of patients used PIM-OSHAAs. Murphy et al. reported that over half of the community-dwelling patients diagnosed with AD aged 50 years and above were prescribed at least one PIM.36 The difference in study findings could be that our study focused on patients with AD aged 65 years and older with insomnia.

In addition, our study observed that among the EI cohort, patients with PIM-OSHAA use had higher HCRU than those without, especially with respect to inpatient stays, inpatient visits, ambulatory care and outpatient visits, ER visits, and pharmacy visits, after adjusting for clinical and sociodemographic covariates. It is worth noting that very few studies have investigated the HCRU associated with PIM-OSHAA use among patients with insomnia, AD, or related disorders. Wickwire et al. evaluated the impact of insomnia treatment (benzodiazepines, trazodone, and zolpidem) on HCRU and costs by comparing outcomes across various treatment groups and found that elderly with insomnia and benzodiazepines treatment experienced increased HCRU, particularly pharmacy visits [7]. The observed differences are possibly attributable to differences in the study design utilized. Notably, our study included zolpidem and benzodiazepines within the PIM-OSHAA group. These study findings imply a continuing healthcare burden due to the prescription of benzodiazepines and other PIM-OSHAAs among the elderly with insomnia.

Similarly, within the ADI cohort, patients with PIM-OSHAA use had more HCRU than those without. These results are consistent with the findings presented by Murphy et al., which showcased that increased PIM use was associated with a greater risk of hospitalizations and outpatient visits [38].

Among both EI and ADI cohorts, patients with PIM-OSHAA use had higher total healthcare costs than those without. There is limited research that has assessed the economic burden associated with PIM-OSHAA among the elderly with insomnia. However, Roldan et al. found similar findings and reported that total healthcare costs for patients treated with PIMs were higher as compared to those without [39]. Another study reported that patients with insomnia who were treated with benzodiazepines had higher healthcare costs [37]. In light of our study findings, prescribing appropriate medications to patients with insomnia is critical to manage their condition and prevent associated high healthcare costs.

The findings of our study should be interpreted considering the inherent limitations associated with claims data. Claims are primarily intended for administrative purposes associated with reimbursement and are subject to coding errors. The study population included those with a diagnosis code for insomnia in claims data. This may not be fully representative of insomnia population in the real-world since insomnia is generally under-reported and might be considered as a differential diagnosis with other sleep disorders. Moreover, coding errors and the use of codes to rule out diagnoses can lead to misclassification of disease. In this study, non-insomnia sleep disorders were excluded; however, those with Rapid Eye Movement sleep behavior disorder (G47.52) or parasomnia, unspecified (G47.50) were not excluded in the cohort identification process. The proportion of those with comorbid G47.50 or G47.52 was < 1%; thus, this error was considered trivial. Pharmacy claims represent prescription filling but cannot ensure the medication was taken as prescribed. In addition, the study sample required continuous enrollment for at least 1-year post-insomnia diagnosis, potentially limiting results generalizability to patients who have survived that period.

Furthermore, the database contains only claims from commercial insurance and Medicare supplemental. Therefore, patients with insomnia and AD who are likely to be covered by only Medicare fee-for-service plans may be excluded [40]. Data limitations regarding the cost components may also exist. For instance, services may not be captured if Medicare is the sole payer or variations in health plan reimbursement. Therefore, the cost estimates in this study may be underestimated. The financial estimates in this study reflect the payer perspective, and the actual out-of-pocket prices paid by patients for healthcare were unavailable. Despite adjusting for sociodemographic and clinical characteristics, residual confounding may persist. While using a large sample size could be considered as a strength of the study in terms of generalizability of the findings, *p*-values should be interpreted with caution. Due to the large sample size, there is a possibility for overestimation of the difference between nearly identical values. Finally, operational definitions could vary across observational studies using administrative databases. Based on the FDA-provided list, this study did not include some commonly prescribed medications used to treat insomnia such as trazodone. Further research is warranted to address the limitations of the study.

## Conclusions

Among elderly patients with insomnia, substantial PIM-OSHAA utilization was observed, particularly among those with comorbid AD. Based on Beers criteria, some OSHAA medications (such as DORAs) were not deemed inappropriate in the elderly; however, those medications were observed to be underutilized. Concerted efforts are needed to increase the utilization of appropriate medications for insomnia treatment in the elderly considering the Beers list. Moreover, higher HCRU and costs were associated with PIM-OSHAA use among the EI and ADI cohorts. This underscores the need to prioritize appropriate medications for insomnia management to improve the quality of care in the elderly population.

## Supplementary material

Supplementary material is available at *SLEEP* online.

zsaf003_suppl_Supplementary_Tables_S1-S7_Figures_S1-S4

## Data Availability

The data supporting the findings of this study are commercially available from the MERATIVE MarketScan Commercial Claims and Encounters and Medicare Supplemental Database. The availability of data is restricted, as it was used under license for the current research and is not publicly available. Information regarding the datasets analyzed during the current study is available on reasonable request by contacting the corresponding author.
