# Supplementary material for: Utilization of potentially inappropriate sedative-hypnotic and atypical antipsychotic medications among elderly individuals with insomnia and Alzheimer’s disease
Source: Sleep. 2025 Jan 25;48(4):zsaf003. doi: 10.1093/sleep/zsaf003 (PMC11985390; doi:10.1093/sleep/zsaf003)

**Supplementary Tables 1-7**

**Supplementary Figures 1-4**

**Utilization of Potentially Inappropriate Sedative-Hypnotic and Atypical Antipsychotic Medications among Elderly Individuals with Insomnia and Alzheimer’s Disease**

Farid Chekani^1^, Kirti Mirchandani^2^, Saba Zaki^2^, Swarnali Goswami^2^, Manvi Sharma^2^

^1^Merck & Co., Inc., Rahway, NJ, USA

^2^ Complete HEOR Solutions (CHEORS), Chalfont, PA, USA

**Corresponding Author**: Farid Chekani, Merck & Co., Inc., 351, North Sumneytown Pike, North Wales, PA, USA, Phone no.: 215-630-3884, E-mail: [farid.chekani@merck.com](mailto:farid.chekani@merck.com)

Supplementary Table 1: ICD-10 code list for AD, Insomnia, NPS subtypes, schizophrenia, and bipolar disorder identification

| **Disease** | **Sub-types** | **ICD-10 Code** | **Description** |
| --- | --- | --- | --- |
| Alzheimer's disease | Alzheimer's disease | G30.0 | Alzheimer's Disease with early onset |
|  |  | G30.1 | Alzheimer's Disease with late onset |
|  |  | G30.8 | Other Alzheimer's disease |
|  |  | G30.9 | Alzheimer's disease, unspecified |
| Insomnia | Insomnia | F51.XX, G47.XX | Insomnia |
| **Neuropsychiatric Symptoms** | | | |
| Psychotic Symptoms | Delusion | F22 | Delusional disorders |
|  | Hallucination | R44x | Other symptoms and signs involving general sensations and perceptions |
|  |  | F06.0 | Psychotic disorder with hallucinations due to known physiological condition |
|  | Other psychotic symptoms | F06.2 | Psychotic disorder with delusions due to known physiological condition |
|  |  | F23 | Brief psychotic disorder |
|  |  | F24 | Shared psychotic disorder |
|  |  | F28 | Other psychotic disorders not due to a substance or known physiological condition |
|  |  | F29 | Unspecified psychosis not due to a substance or known physiological condition |
| Agitation/Aggression | Agitation | R45.1 | Restlessness and agitation |
|  |  | R46.3 | Overactivity |
|  | Aggression | R45.6 | Violent behavior |
|  |  | F91.8 | Other conduct disorders |
|  | Irritability | R45.4 | Irritability and anger |
|  | Sexual disinhibition | F03.A18 |  |
|  | Wandering | Z91.83 | Wandering in diseases classified elsewhere |
| Anxiety/Mood disorders | Anxiety | F41.0,  F41.1,  F41.3,  F41.8,  F41.9 | Other anxiety disorders |
|  | Mood    disorders | R45.3 | Apathy |
|  |  | F34.8x | Other persistent mood [affective] disorders |
|  |  | F39.x | Unspecified Mood (affective) disorder |
|  | Depression/Dysphoria | F32.x | Depressive episode |
|  |  | F33.x | Major depressive disorder, recurrent |
|  |  | F34.1 | Dysthymic disorder |
|  |  | F41.2 | Mixed anxiety and depressive disorder |
| **Disease conditions to be excluded** | | | |
| Schizophrenia | Schizophrenia | F20.0 | Paranoid schizophrenia |
|  |  | F20.1 | Disorganized schizophrenia |
|  |  | F20.2 | Catatonic schizophrenia |
|  |  | F20.3 | Undifferentiated schizophrenia |
|  |  | F20.5 | Residual schizophrenia |
|  |  | F20.8 | Other schizophrenia |
|  |  | F20.81 | Disorganized schizophrenia |
|  |  | F20.89 | Other schizophrenia |
|  |  | F20.9 | Schizophrenia, unspecified |
|  |  | F25 | Schizoaffective disorders |
|  |  | F25.0 | Schizoaffective disorder, bipolar type |
|  |  | F25.1 | Schizoaffective disorder, depressive type |
|  |  | F25.8 | Other schizoaffective disorders |
|  |  | F25.9 | Schizoaffective disorder, unspecified |
| Bipolar Disorder | Manic or mixed-type bipolar disorder | F30.x | Manic Episode |
|  |  | F31.10 | Bipolar disorder, current episode manic without psychotic features, unspecified |
|  |  | F31.11 | Bipolar disorder, current episode manic without psychotic features, mild |
|  |  | F31.12 | Bipolar disorder, current episode manic without psychotic features, moderate |
|  |  | F31.13 | Bipolar disorder, current episode manic without psychotic features, severe |
|  |  | F31.2 | Bipolar disorder, current episode manic severe with psychotic features |
|  |  | F31.60 | Bipolar disorder, current episode mixed, unspecified |
|  |  | F31.61 | Bipolar disorder, current episode mixed, mild |
|  |  | F31.62 | Bipolar disorder, current episode mixed, moderate |
|  |  | F31.63 | Bipolar disorder, current episode mixed, severe, without psychotic features |
|  |  | F31.64 | Bipolar disorder, current episode mixed, severe, with psychotic features |
|  |  | F31.73 | Bipolar disorder, in partial remission, most recent episode manic |
|  |  | F31.74 | Bipolar disorder, in full remission, most recent episode manic |
|  |  | F31.77 | Bipolar disorder, in partial remission, most recent episode mixed |
|  |  | F31.78 | Bipolar disorder, in full remission, most recent episode mixed |

Supplementary Table 2: List of insomnia medications

| **Therapeutic Class** | **Drug** |
| --- | --- |
| Atypical antipsychotics (AAPs) | Aripiprazole (Abilify) |
|  | Asenapine (Saphris) |
|  | Brexpiprazole (Rexulti) |
|  | Cariprazine (Vraylar) |
|  | Clozapine (Clozaril) |
|  | Iloperidone (Fanapt) |
|  | Lurasidone (Latuda) |
|  | Olanzapine (Zyprexa) |
|  | Paliperidone (Invega) |
|  | Quetiapine (Seroquel) |
|  | Risperidone (Risperdal) |
|  | Ziprasidone (Geodon) |
| Dual Orexin Receptor Antagonist | Lemborexant |
|  | Daridorexant |
|  | Suvorexant |
| Benzodiazepine | Flurazepam |
|  | Temazepam |
|  | Triazolam |
|  | Estazolam |
|  | Quazepam |
| Z-drugs | Zolpidem |
|  | Zaleplon |
|  | Eszopiclone |
| Melatonin Agonist | Ramelteon |
|  | Tasimelteon |
| Tricyclic Antidepressant | Doxepin |

*It was assumed that any non-oral formulations of drugs (cream, jelly/gel, or powder) might not be prescribed for insomnia treatment, therefore, the related claims were excluded.

Supplementary Table 3: Code list for non-insomnia sleep disorders

| **Description** | **ICD-10 codes** |
| --- | --- |
| Hypersomnia | G47.10, G47.11, G47.1, G47.12, G47.13, G47.14, G47.19, F51.11, F51.12, F51.13, F51.19 |
| Parasomnia | F51.3, F51.4, F51.5 |
| Obstructive sleep apnea | G47.33, G47.3, G47.30, G47.30 |
| Narcolepsy | G47.4, G47.411, G47.419, G47.421, G47.429 |
| Restless Legs Syndrome | G25.8 |
| Circadian Rhythm Sleep-Wake disorders | F51.2, G47.2 |

Supplementary Table 4 : Additional baseline characteristics of patients with or without PIM-OSHAA use among EI cohort

| **Description** | **PIM-OSHAA** | **No PIM-OSHAA** | **p-Value** |
| --- | --- | --- | --- |
|  | **(N=30,705)** | **(N=122,264)** |  |
| **Region N (%)** | | | |
| North central | 6,943 (22.61%) | 36,146 (29.56%) | **<.001** |
| Northeast | 8,895 (28.97%) | 33,945 (27.76%) |  |
| Other | 48 ( 0.16%) | 186 ( 0.15%) |  |
| South | 11,392 (37.10%) | 38,908 (31.82%) |  |
| West | 3,427 (11.16%) | 13,079 (10.70%) |  |
| **Year of Index date N (%)** | | | |
| 2017 | 12,081 (39.35%) | 40,125 (32.82%) | **<.001** |
| 2018 | 7,954 (25.90%) | 28,493 (23.30%) |  |
| 2019 | 4,468 (14.55%) | 19,561 (16.00%) |  |
| 2020 | 2,200 ( 7.16%) | 10,823 ( 8.85%) |  |
| 2021 | 4,002 (13.03%) | 23,262 (19.03%) |  |
| **Plan type N (%)** | | | |
| Comprehensive | 5,933 (19.32%) | 24,306 (19.88%) | **<.001** |
| HMO | 3,004 ( 9.78%) | 15,167 (12.41%) |  |
| PPO | 18,308 (59.63%) | 69,849 (57.13%) |  |
| Other | 3,186 (10.38%) | 11,528 ( 9.43%) |  |
| Missing | 274 ( 0.89%) | 1,414 ( 1.16%) |  |
| **Comorbidities**   **N (%)** | | | |
| Congestive heart failure | 2,957 ( 9.63%) | 14,397 (11.78%) | **<.001** |
| Cardiac arrhythmias | 6,762 (22.02%) | 30,887 (25.26%) | **<.001** |
| Valvular disease | 4,347 (14.16%) | 18,317 (14.98%) | **<.001** |
| Pulmonary circulation disorders | 969 ( 3.16%) | 5,018 ( 4.10%) | **<.001** |
| Peripheral vascular disorders | 4,581 (14.92%) | 18,845 (15.41%) | **0.032** |
| Hypertension, uncomplicated | 19,660 (64.03%) | 82,685 (67.63%) | **<.001** |
| Hypertension, complicated | 3,227 (10.51%) | 15,182 (12.42%) | **<.001** |
| Paralysis | 331 ( 1.08%) | 1,487 ( 1.22%) | **0.046** |
| Other neurological disorders | 2,894 ( 9.43%) | 9,824 ( 8.04%) | **<.001** |
| Chronic pulmonary disease | 6,182 (20.13%) | 25,643 (20.97%) | **<.001** |
| Diabetes, uncomplicated | 6,510 (21.20%) | 31,686 (25.92%) | **<.001** |
| Diabetes, complicated | 4,393 (14.31%) | 22,343 (18.27%) | **<.001** |
| Hypothyroidism | 6,427 (20.93%) | 23,235 (19.00%) | **<.001** |
| Renal failure | 3,170 (10.32%) | 13,970 (11.43%) | **<.001** |
| Liver disease | 1,604 ( 5.22%) | 6,653 ( 5.44%) | 0.131 |
| Peptic ulcer disease excluding bleeding | 393 ( 1.28%) | 1,203 ( 0.98%) | **<.0001** |
| AIDS/HIV | 57 ( 0.19%) | 140 ( 0.11%) | **0.0019** |
| Lymphoma | 451 ( 1.47%) | 1,591 ( 1.30%) | **0.022** |
| Metastatic cancer | 650 ( 2.12%) | 1,892 ( 1.55%) | **<.001** |
| Solid tumor without metastasis | 3,766 (12.27%) | 14,585 (11.93%) | 0.105 |
| Rheumatoid arthritis/collagen vascular diseases | 2,370 ( 7.72%) | 7,915 ( 6.47%) | **<.001** |
| Coagulopathy | 1,179 ( 3.84%) | 4,829 ( 3.95%) | 0.376 |
| Obesity | 3,821 (12.44%) | 22,580 (18.47%) | **<.001** |
| Weight loss | 1,236 ( 4.03%) | 3,969 ( 3.25%) | **<.001** |
| Fluid and electrolyte disorders | 3,654 (11.90%) | 13,421 (10.98%) | **<.001** |
| Blood loss anemia | 482 ( 1.57%) | 1,902 ( 1.56%) | 0.858 |
| Deficiency anemia | 2,183 ( 7.11%) | 7,930 ( 6.49%) | **<.001** |
| Alcohol abuse | 603 ( 1.96%) | 1,851 ( 1.51%) | **<.001** |
| Drug abuse | 737 ( 2.40%) | 1,514 ( 1.24%) | **<.001** |

Notes: EI: Elderly with insomnia; HMO: Health Maintenance Organization; PIM-OSHAA: Inappropriate Oral Sedative Hypnotics; PPO: Preferred provider organizations.

^a^*p-*Value compares differences between PIM-OSHAA and no PIM-OSHAA group. Significant *p-*Values are marked as bold

Supplementary Table 5 : Additional baseline characteristics of patients with or without PIM-OSHAA use among ADI cohort

| **Description** | **PIM-OSHAA** | **No PIM-OSHAA** | **p-Value** |
| --- | --- | --- | --- |
|  | **(N=1,728)** | **(N=3,160)** |  |
| **Region N (%)** | | | |
| North central | 486 (28.13%) | 1,069 (33.83%) | **<0.001** |
| Northeast | 536 (31.02%) | 879 (27.82%) |  |
| Other | - | - |  |
| South | 562 (32.52%) | 937 (29.65%) |  |
| West | 144 ( 8.34%) | 275 ( 8.70%) |  |
| **Year of Index date N (%)** | | | |
| 2017 | 600 (34.72%) | 1,092 (34.56%) | 0.854 |
| 2018 | 377 (21.82%) | 696 (22.03%) |  |
| 2019 | 260 (15.05%) | 450 (14.24%) |  |
| 2020 | 115 ( 6.66%) | 233 ( 7.37%) |  |
| 2021 | 376 (21.76%) | 689 (21.80%) |  |
| **Plan type N (%)** | | | |
| Comprehensive | 532 (30.79%) | 1,003 (31.74%) | 0.574 |
| HMO | 91 ( 5.27%) | 197 ( 6.23%) |  |
| PPO | 1,032 (59.72%) | 1,837 (58.13%) |  |
| Other | 57 ( 3.30%) | 97 ( 3.07%) |  |
| Missing | 16 ( 0.93%) | 26 ( 0.82%) |  |
| **Comorbidities**   **N (%)** | | | |
| Congestive heart failure | 286 (16.55%) | 656 (20.76%) | **<0.001** |
| Cardiac arrhythmias | 609 (35.24%) | 1,206 (38.16%) | **0.043** |
| Valvular disease | 336 (19.44%) | 577 (18.26%) | 0.310 |
| Pulmonary circulation disorders | 83 ( 4.80%) | 159 ( 5.03%) | 0.725 |
| Peripheral vascular disorders | 511 (29.57%) | 947 (29.97%) | 0.772 |
| Hypertension, uncomplicated | 1,332 (77.08%) | 2,431 (76.93%) | 0.903 |
| Hypertension, complicated | 317 (18.34%) | 613 (19.40%) | 0.370 |
| Paralysis | 43 ( 2.49%) | 80 ( 2.53%) | 0.927 |
| Other neurological disorders | 613 (35.47%) | 1,140 (36.08%) | 0.675 |
| Chronic pulmonary disease | 346 (20.02%) | 668 (21.14%) | 0.358 |
| Diabetes, uncomplicated | 436 (25.23%) | 833 (26.36%) | 0.389 |
| Diabetes, complicated | 314 (18.17%) | 649 (20.54%) | **0.047** |
| Hypothyroidism | 427 (24.71%) | 735 (23.26%) | 0.255 |
| Renal failure | 304 (17.59%) | 609 (19.27%) | 0.150 |
| Liver disease | 46 ( 2.66%) | 124 ( 3.92%) | **0.021** |
| Peptic ulcer disease excluding bleeding | 30 ( 1.74%) | 40 ( 1.27%) | 0.189 |
| AIDS/HIV | 0 (0.0) | 2 ( 0.06%) | 0.543 |
| Lymphoma | 24 ( 1.39%) | 44 ( 1.39%) | 0.992 |
| Metastatic cancer | 26 ( 1.50%) | 38 ( 1.20%) | 0.374 |
| Solid tumor without metastasis | 189 (10.94%) | 344 (10.89%) | 0.956 |
| Rheumatoid arthritis/collagen vascular diseases | 93 ( 5.38%) | 216 ( 6.84%) | **0.046** |
| Coagulopathy | 89 ( 5.15%) | 181 ( 5.73%) | 0.398 |
| Obesity | 136 ( 7.87%) | 293 ( 9.27%) | 0.098 |
| Weight loss | 210 (12.15%) | 395 (12.50%) | 0.725 |
| Fluid and electrolyte disorders | 433 (25.06%) | 791 (25.03%) | 0.984 |
| Blood loss anemia | 38 ( 2.20%) | 81 ( 2.56%) | 0.430 |
| Deficiency anemia | 185 (10.71%) | 319 (10.09%) | 0.502 |
| Alcohol abuse | 43 ( 2.49%) | 55 ( 1.74%) | 0.075 |
| Drug abuse | 26 ( 1.50%) | 33 ( 1.04%) | 0.159 |

Notes: ADI: Elderly with Alzheimer’s disease and insomnia; HMO: Health Maintenance Organization; PIM-OSHAA: Inappropriate Oral Sedative Hypnotics; PPO: Preferred provider organizations.

^a^*p-*Value compares differences between PIM-OSHAA and no PIM-OSHAA group. Significant *p-*Values are marked as bold

**Sensitivity Analysis: EI cohort**

Supplementary Table 6 : Comparison of all-cause HCRU and healthcare costs between patients with or without PIM-OSHAA use: EI cohort (Weighted Regression Analysis)

| **Categories** | **OR [95% CI]** | **p-Value** | **IRR [95% CI]** | **p-Value** |  |
| --- | --- | --- | --- | --- | --- |
| **HCRU** | | | | |  |
| Inpatient LOS (in days) | 1.15 [1.13,1.17] | **<0.001** | 1.18 [1.16,1.21] | **<0.001** |  |
| Inpatient visits | 1.15 [1.12,1.17] | **<0.001** | 1.05 [1.04,1.06] | **<0.001** |  |
| Ambulatory Care & Outpatient visits | One part |  | 1.07 [1.07,1.08] | **<0.001** |  |
| Hospice visits | 0.99 [0.86,1.13] | 0.836 | 0.70 [0.58,0.85] | **<0.001** |  |
| Other medical claims | One part |  | 0.99 [0.98,1.01] | 0.343 |  |
| ER visits | 1.12 [1.10,1.14] | **<0.001** | 1.10 [1.09,1.11] | **<0.001** |  |
| Physician visits | One part |  | 1.02 [1.01,1.04] | **<0.001** |  |
| Pharmacy visits | One part |  | 1.28 [1.28,1.28] | **<0.001** |  |
| **Healthcare Cost** |  |  |  |  |  |
| Total costs | One part |  | 1.14 [1.12,1.15] | **<0.001** |  |
| Inpatient costs | 1.15 [1.13,1.17] | **<0.001** | 1.05 [1.03,1.08] | **<0.001** |  |
| Ambulatory Care & Outpatient costs | One part |  | 1.11 [1.09,1.12] | **<0.001** |  |
| Hospice costs | 0.96 [0.84,1.11] | 0.584 | 0.81 [0.60, 1.09] | 0.169 |  |
| Other medical claims costs | One part |  | 1.11 [1.08,1.14] | **<0.001** |  |
| ER costs | 1.12 [1.10,1.14] | **<0.001** | 1.11 [1.08, 1.13] | **<0.001** |  |
| Physician costs | One part |  | 1.11 [1.08,1.13] | **<0.001** |  |
| Pharmacy costs | One part |  | 1.17 [1.15,1.19] | **<0.001** |  |

Notes: CI: Confidence Interval; EI: Elderly with insomnia; ER: Emergency Room; IRR: Incidence rate ratio; HCRU: Healthcare resource utilization; LOS: Length of stay; OR: Odds ratio; PIM-OSHAA: Potentially Inappropriate Oral Sedative Hypnotic Medication

^a^ OR and IRR are for patients with PIM-OSHAA use relative to those without. *p-*Values less than 0.05 are significant and are marked in bold.

**Sensitivity Analysis: ADI cohort**

Supplementary Table 7: Comparison of all-cause HCRU and healthcare costs between patients with or without PIM-OSHAA use among ADI cohort (Weighted Regression Analysis)

| **Categories** | **OR [95% CI]** | ***P* Value** | **IRR [95% CI]** | ***P* Value** |  |
| --- | --- | --- | --- | --- | --- |
| **HCRU** | | | | |  |
| Inpatient LOS (in days) | 1.55 [1.42,1.68] | **<0.001** | 1.18 [1.08,1.28] | **<0.001** |  |
| Inpatient visits | 1.51 [1.40,1.64] | **<0.001** | 1.05 [1.01,1.10] | **<0.001** |  |
| Ambulatory Care & Outpatient visits | One part |  | 1.07 [1.03,1.11] | **0.001** |  |
| Hospice visits | 1.05 [0.71,1.55] | 0.825 | 0.63 [0.43,0.92] | **0.016** |  |
| Other medical claims | One part |  | 1.15[1.08,1.22] | **<0.001** |  |
| ER visits | 1.59 [1.47,1.71] | **<0.001** | 1.13 [1.06,1.19] | **<0.001** |  |
| Physician visits | One part |  | 0.95 [0.88,1.01] | 0.099 |  |
| Pharmacy visits | One part |  | 1.16 [1.12,1.20] | **<0.001** |  |
| **Healthcare Cost** |  |  |  |  |  |
| Total costs | One part |  | 1.13 [1.06,1.21] | **<0.001** |  |
| Inpatient costs | 1.53 [1.41,1.66] | **<0.001** | 1.03 [0.93,1.14] | **0.605** |  |
| Ambulatory Care & Outpatient costs | One part |  | 0.90 [0.84,0.96] | **0.001** |  |
| Hospice costs | 0.93 [0.62,1.40] | 0.735 | 0.56 [0.55, 0.56] | **<0.001** |  |
| Other medical claims costs | One part |  | 1.01 [0.89,1.14] | 0.924 |  |
| ER costs | 1.57 [1.45,1.70] | **<0.001** | 1.04 [0.94, 1.16] | 0.449 |  |
| Physician costs | One part |  | 0.85 [0.74,0.97] | **0.017** |  |
| Pharmacy costs | One part |  | 1.02 [0.94,1.11] | 0.578 |  |

Notes: ADI: Elderly with Alzheimer’s disease and insomnia; CI: Confidence Interval; ER: Emergency Room; IRR: Incidence rate ratio; HCRU: Healthcare resource utilization; LOS: Length of stay; OR: Odds ratio; PIM-OSHAA: Potentially Inappropriate Oral Sedative Hypnotic Medication

^a^ OR and IRR are for patients with PIM-OSHAA use relative to those without. *p-*Values less than 0.05 are significant and are marked in bold.


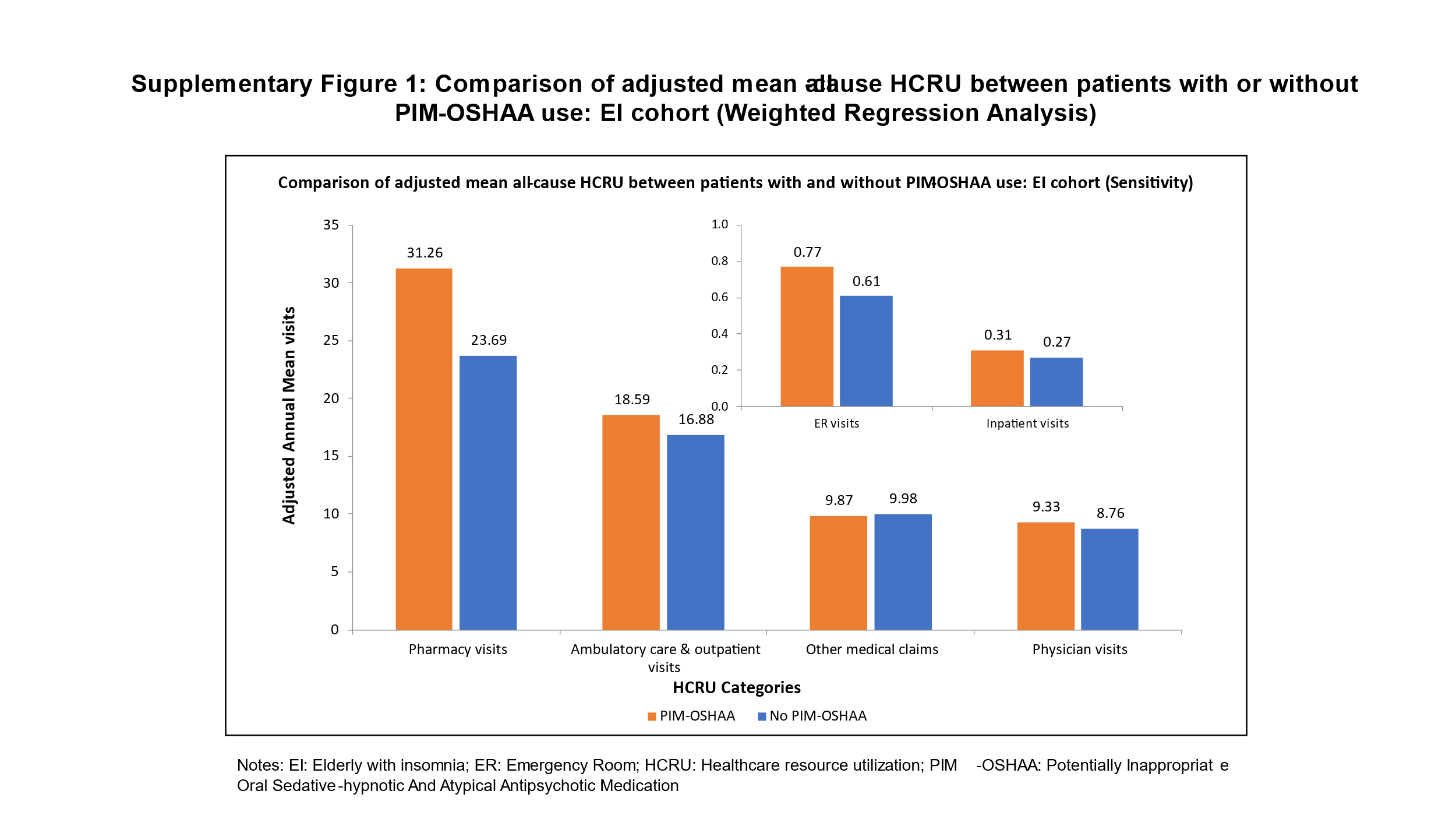


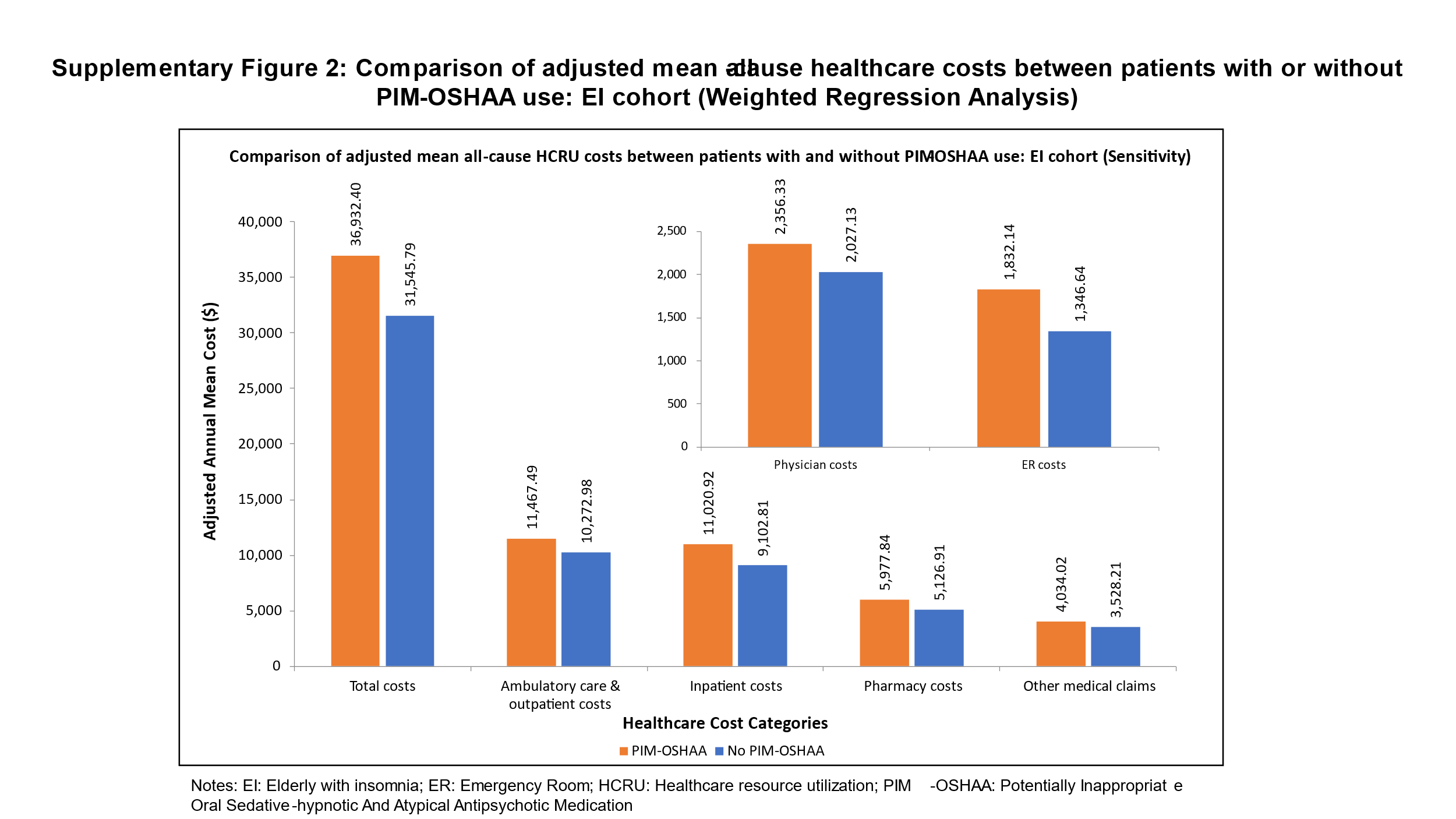


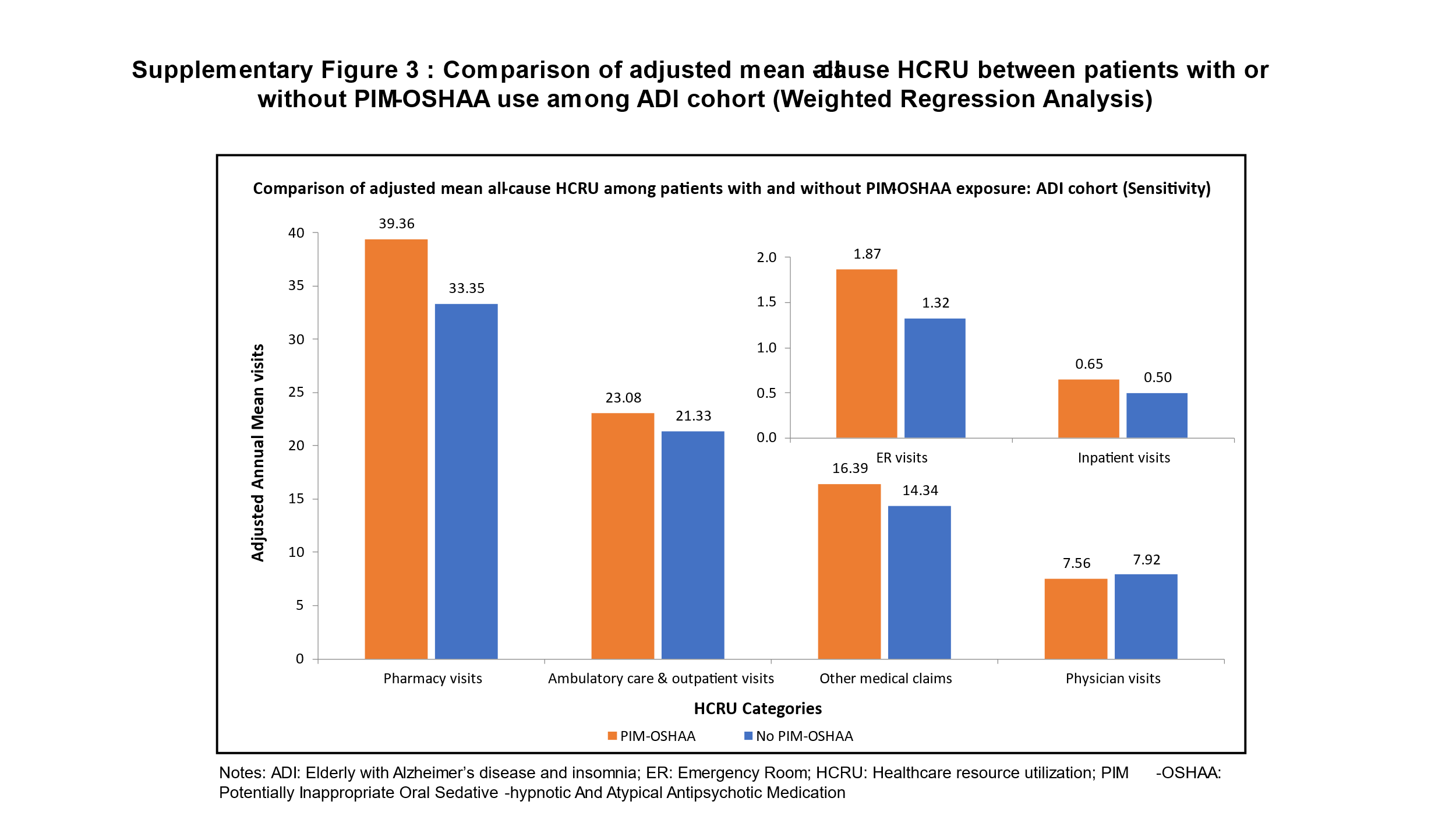


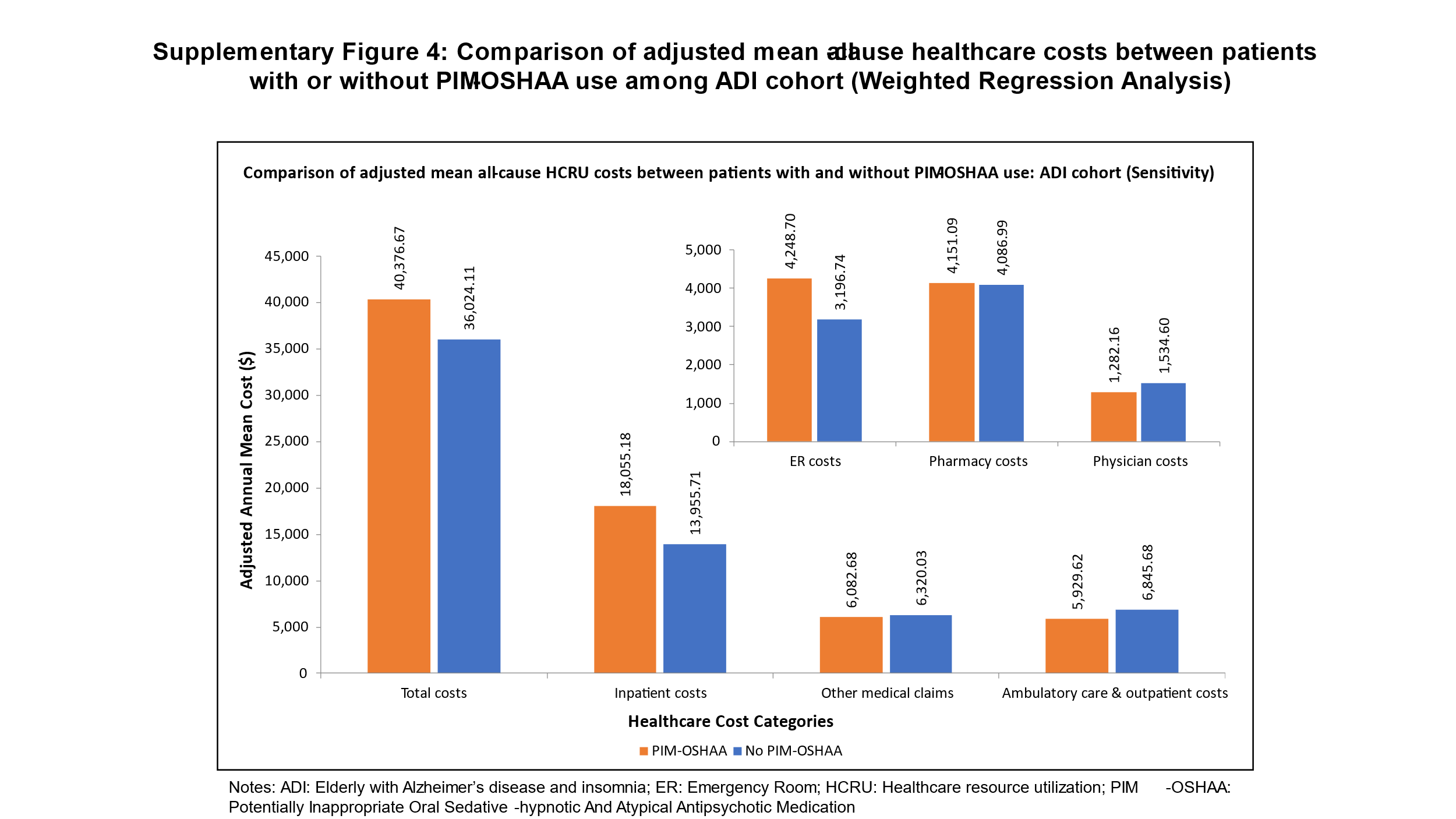

Supplement: zsaf003_suppl_Supplementary_Tables_S1-S7_Figures_S1-S4 [file zsaf003_suppl_supplementary_tables_s1-s7_figures_s1-s4.docx]
